# Drug repurposing based on differentially expressed genes suggests drug combinations with possible synergistic effects in treatment of lung adenocarcinoma

**DOI:** 10.1080/15384047.2023.2253586

**Published:** 2023-09-14

**Authors:** Liora Yesharim, Shahram Teimourian

**Affiliations:** Department of Medical Genetics, School of Medicine, Iran University of Medical Sciences, Tehran, Iran

**Keywords:** Lung adenocarcinoma, drug repurposing, drug repositioning, hub genes, meta-analysis, differentially expressed genes

## Abstract

Lung adenocarcinoma is one of the leading causes of cancer-related mortality globally. Various treatment approaches and drugs had little influence on overall survival; thus, new drugs and treatment strategies are needed. Drug repositioning (repurposing) seems a favorable approach considering that developing new drugs needs much more time and costs. We performed a meta-analysis on 6 microarray datasets to obtain the main genes with significantly altered expression in lung adenocarcinoma. Following that, we found major gene clusters and hub genes. We assessed their enrichment in biological pathways to get insight into the underlying biological process involved in lung adenocarcinoma pathogenesis. The L1000 database was explored for drug perturbations that might reverse the expression of differentially expressed genes in lung adenocarcinoma. We evaluated the potential drug combinations that interact the most with hub genes and hence have the most potential to reverse the disease process. A total of 2148 differentially expressed genes were identified. Six main gene clusters and 27 significant hub genes mainly involved in cell cycle regulation have been identified. By assessing the interaction between 3 drugs and hub genes and information gained from previous clinical investigations, we suggested the three possible repurposed drug combinations, Vorinostat – Dorsomorphin, PP-110 – Dorsomorphin, and Puromycin – Vorinostat with a high chance of success in clinical trials.

## Introduction

Lung cancer is the second most common cancer and the leading cause of cancer-related death worldwide.^1^ Lung adenocarcinoma (LUAD) is the main histologic subtype of non-small cell lung cancer (NSCLC) and accounts for 40% of all lung cancers. Adenocarcinoma of the lung usually evolves from mucosal glands and is common in both smokers and nonsmokers.^2^ Unfortunately, most patients are diagnosed at late stages, which results in a low survival rate and limited treatment options.^3,4^

Three general drug classes are used to treat patients individually or in combination: chemotherapy, targeted therapy, and immunotherapy. The most commonly used drugs in chemotherapy are platinum-based drugs such as carboplatin and cisplatin. However, extensive side effects of chemotherapy, such as neurotoxicity, thrombocytopenia, and renal toxicity, prompted the development and usage of targeted therapies.^5,6^ Targeted therapy is prescribed based on patients’ mutation profiles. Antiangiogenic agents like bevacizumab and ramucirumab and tyrosine kinase inhibitors such as erlotinib and gefitinib are among the most significant target-specific therapeutics.^7,8^ Practically, targeted therapy is only beneficial for a small number of patients as not all mutations can be targeted, also drug resistance occurs after a period of treatment.^3,9^ Therefore, immunotherapy was developed to combat drug resistance. However, there are certain disadvantages to this therapeutic approach too. Aside from their limited effectiveness, drug resistance to these medications has also been observed.^10^ Even worse, it has been revealed that using some immunotherapy drugs accelerates the spread of cancer.^11^ Despite all the efforts and the use of numerous therapies and medications, around 1.8 million individuals died from lung cancer in 2020, making it the deadliest cancer.^1^ So new drugs and efficient therapeutic approaches are desperately needed. However, the design and development of new pharmaceuticals take a long time and much money before they are authorized as safe and effective medicines. Repurposing current drugs is a feasible approach that can help meet the need for more effective therapeutics. Drug repurposing (aka drug repositioning) identifies new therapeutic indications for existing drugs, allowing faster lead times, and lower risks and costs than developing new drugs.^12^

We employed differentially expressed genes (DEGs) in lung adenocarcinoma (LUAD) obtained by meta-analysis of GEO microarray datasets to establish the gene signature of (LUAD) pathogenesis. Then, by utilizing LINCS L1000 drug perturbation data sets,^13^ we identified and selected small molecules with the potential to reverse tumor cell expression patterns. Following that, we investigated the interaction and regulatory role of the discovered small molecules with DEGs and hub genes to clarify their probable mechanism of action.

## Methods

### Microarray data selection

A total of six publicly available datasets (GSE85841, part of GSE103512, GSE116959, GSE118370, GSE136043, GSE14797) related to LUAD which includes 150 samples (40 normal and 110 cancers) were downloaded from GEO (https://www.ncbi.nlm.nih.gov/geo/) database and entered into R software^14^ (version 4.0.0) for quality control and outlier removal. All six datasets had at least 10 samples of human LUAD and adjacent non-tumor tissue. GSE85841 and GSE118370 were quantile normalized using the R package “limma”^15^ to minimize technical variation. (The other four datasets were already normalized and did not require any further normalization.) By using a PCA plot, three samples were identified as outliers and were removed.

### Gene expression meta-analysis and DEGs identification

**Figure 1.**
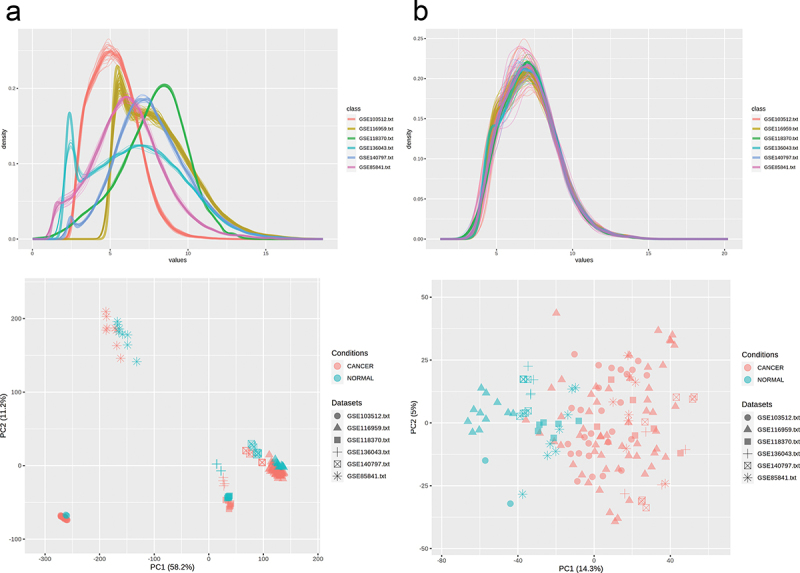
The density and PCA plot of the six microarray datasets, (a) before and (b) after batch effect removal.

**Figure 2.**
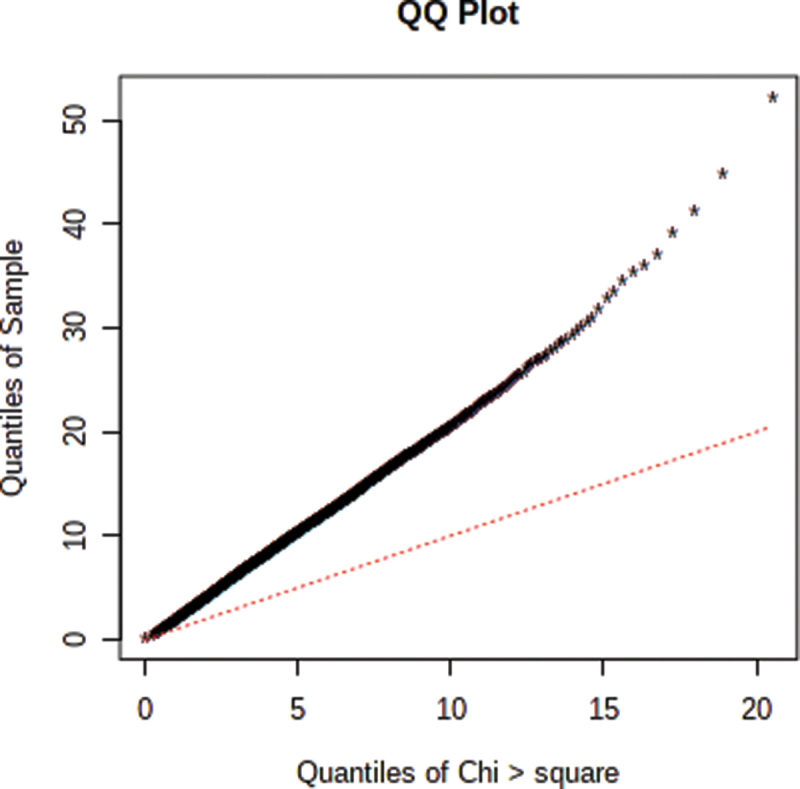
Q-Q plot drawn after submitting the six datasets to (www.networkanalyst.ca). The red line indicates the chi-squared distribution. This Q-Q plot shows that the distribution of the datasets used for meta-analysis is different.

Meta-analysis improves statistical power and data validity. The NetworkAnalyst web interface was used for microarray meta-analysis.^16,17^ The six datasets, totaling 147 samples, were submitted to NetworkAnalyst. Differential expression analysis was performed on six datasets individually using limma with the cutoff of 0.01 Benjamini-Hochberg’s adjusted p-value and |log_2_FC|>1. The batch effect was adjusted using the ComBat function in R package named SVA.^18^ Batch effect removal is necessary in order to remove variations that happen unrelated to biological differences. Figure 1 shows samples clustering patterns before and after batch effect removal. The differential expression meta-analysis of LUAD and normal tissues was carried out using the combined effect size. As Cochrane’s Q test (Figure 2) showed significant deviation from a chi-squared distribution, the random effect model (REM) was chosen over the fixed effect model (FEM).

### Functional and pathway enrichment analysis

Gene Ontology (GO) enrichment analysis is the most common way for the extraction of meaningful information and classification of genes obtained from high-throughput data based on their biological process (BP), cellular component (CC), and molecular function (MF). Pathway enrichment analysis is also valuable to gain an understanding of the most enriched biological pathways. GO functional enrichment analysis and KEGG pathway enrichment analysis of DEGs were conducted using the Database for Annotation, Visualization, and Integrated Discovery (DAVID) (http://david.abcc.ncifcrf.gov/). We uploaded up-regulated and down-regulated genes separately. We used the lists of merged genes used in the meta-analysis as background. P-value ≥.01 was set as the cutoff criterion.

### Constructing the PPI network and module analysis

A protein-protein interaction (PPI) network of all 2963 DEGs (not considering log_2_FC cutoff for DEGs) was established using the Cytoscape^19^ STRING app^20^ (version 1.6.0). The PPI network was constructed and visualized by Cytoscape software (version 3.8.2). To reduce the number of interactions to those with higher confidence and a higher possibility of being true positives, we used a confidence cutoff value of 0.95. The full STRING network was incorporated which is based on proteins functional associations. One large network and several smaller networks (most of which had fewer than three nodes) were obtained.

The largest network containing 867 nodes and 2545 edges was chosen for further analysis.

The Cytoscape plugin Molecular Complexity Detection (MCODE) (version 2.0.0) was used to find the most significant module in the largest PPI network.^21^ The module selection criteria are as follows: Degree cutoff = 3, k-core = 5, maximum depth = 100, and node score cutoff = 0.2. KEGG pathway enrichment analysis was performed for the obtained modules.

### Hub genes selection, validation, and prognosis prediction

The top 30 hub genes in the PPI network were identified using the Cytoscape plugin CytoHubba (version 0.1).^22^ The Maximal Clique Centrality (MCC) algorithm was chosen for hub gene selection since it was proven to be the most effective and reliable method of detecting hub nodes.^22^ The 30 hub genes were examined using the GEPIA online tool to validate gene expression levels in LUAD samples vs. normal lung tissues. “GEPIA is an online tool based on the Cancer Genome Atlas (TCGA) and the Genotype-Tissue Expression (GTEx) projects using the output of a standard processing pipeline for RNA sequencing data”.^23^ The significance criterion was chosen to be p-value ≥.01 and |log_2_FC|>1. Overall Survival (OS) analysis was also performed using the Kaplan – Meier method for validated hub genes by using the http://gepia.cancer-pku.cn online web-tool, with the Log-rank (aka the Mantel-Cox) hypothesis test.

### Potential drugs identification

The L1000CDS^2^ search engine was used to identify potential small molecules which can reverse the expression pattern in LUAD. L1000CDS^2^ can rapidly search through the Library of Integrated Cellular Signatures (LINCS) L1000 data set, finding and prioritizing small molecules that can either reverse or mimic the expression signature that users provide.^24^ LINCS L1000 dataset includes the chemically perturbed expression profiles of 62 unique cell lines in response to 3924 different small molecules.^13^ A large number of drug perturbation profiles made LINCs data set a valuable resource for drug repurposing. The submission of up-regulated and down-regulated genes to the L1000CDS^2^ search engine resulted in the list of the top 50 drug perturbations which can reverse the input signature.

In addition, L1000CDS^2^ was investigated for possible effective drug combinations. Drug combinations (a combination of two or more therapeutic agents) appear to be more beneficial for complicated disease therapies. It increases effectiveness by concentrating on redundant biological systems and reducing toxicity by enhancing selectivity and decreasing dosage requirements.^25^ The 50 top drug perturbation signatures were compared pairwise, and a synergy calculation was conducted for each pair. The synergy score was calculated by combining the overlap of the DEGs produced by the two drug treatments with our input DEGs. This search engine compared our input DEGs to the differentially expressed genes already computed from the LINCS L1000 data and returned the top 50 signatures with the largest cosine distances that could potentially reverse the expression of our DEGs. The overlap of the DEGs produced by the two drug treatments was then calculated by comparing the DEGs identified in each treatment to the input DEGs. The synergy score was obtained by multiplying the overlap score with the average connectivity score of the two drugs. This approach allowed us to identify potential drug combinations that could effectively target the underlying pathways and mechanisms involved in LUAD. The rationale for this is that “if two perturbations are orthogonal, then they may impart their overall effect through two independent pathways”.^24^ Top three drug combinations selected to assess their interactions with DEGs and hub genes. We assume that drugs with the most interaction with the hub genes may have the most robust anti-cancer applicability since they influence the gene regulatory network by inhibiting hub genes.

## Results

### DEGs identified by microarray meta-analysis

After a series of processes such as quality control, normalization, outlier removal, and batch effect adjustment, the six datasets were used for meta-analysis. The REM-based meta-analysis identified 2148 genes with |log_2_FC|>1 as DEGs, including 944 up-regulated and 1204 down-regulated genes. Adjusted p-value 0.01 was set as the statistically significant threshold for differentially expressed genes (Benjamini-Hochberg corrected for multiple comparisons). Without taking the log_2_FC cutoff into account, a total of 2963 genes were found to be differentially expressed.

### Functional and pathway enrichment analysis

We used the DAVID online web to analyze 944 up-regulated and 1204 down-regulated genes in lung adenocarcinoma to understand GO and KEGG enriched terms better.

The top five results of each category are summarized in Table 1. The up-regulated genes were mainly enriched in cell cycle and DNA replication related pathways. KEGG’s cancer pathways and extracellular matrix organization were among the most important enriched terms for down-regulated genes.

**Table 1.** Results of DAVID GO and KEGG pathway enrichment analysis for up and downregulated genes in LUAD.

Category	Term	Description	Count	PValue	FDR
**Up-regulated in cancer**					
GO_BP	GO:0006260	DNA replication	33	3.60E–11	1.01E–07
GO_BP	GO:0007067	mitotic nuclear division	41	3.23E–10	4.55E–07
GO_BP	GO:0007062	sister chromatid cohesion	25	7.23E–10	6.78E–07
GO_BP	GO:0051301	cell division	48	3.01E–09	2.11E–06
GO_CC	GO:0000777	condensed chromosome kinetochore	19	1.28E–07	5.36E–05
GO_CC	GO:0005819	spindle	23	2.32E–07	5.36E–05
GO_CC	GO:0000922	spindle pole	21	3.24E–07	5.36E–05
GO_CC	GO:0000776	kinetochore	19	4.11E–07	5.36E–05
GO_CC	GO:0005654	nucleoplasm	200	1.15E–05	0.001070633
GO_BP	GO:0000082	G1/S transition of mitotic cell cycle	23	3.30E–08	1.86E–05
GO_MF	GO:0005524	ATP binding	121	1.61E–05	0.015174774
GO_MF	GO:0008026	ATP-dependent helicase activity	8	9.08E–04	0.273878738
GO_MF	GO:0019901	protein kinase binding	38	0.001114451	0.273878738
GO_MF	GO:0004003	ATP-dependent DNA helicase activity	8	0.001159275	0.273878738
GO_MF	GO:0043021	ribonucleoprotein complex binding	8	0.001823643	0.344668606
KEGG_PATHWAY	hsa04110	Cell cycle	30	1.22E–10	3.03E–08
KEGG_PATHWAY	hsa04115	p53 signaling pathway	12	0.002141067	0.265492334
KEGG_PATHWAY	hsa03410	Base excision repair	8	0.00374774	0.304056415
KEGG_PATHWAY	hsa03460	Fanconi anemia pathway	9	0.004904136	0.304056415
KEGG_PATHWAY	hsa03030	DNA replication	8	0.007430577	0.368556625
**Down-regulated in cancer**					
GO_BP	GO:0030198	extracellular matrix organization	40	2.63E–08	1.07E–04
GO_BP	GO:0001525	angiogenesis	41	1.62E–07	3.31E–04
GO_BP	GO:0043547	positive regulation of GTPase activity	74	4.49E–07	6.11E–04
GO_BP	GO:0007165	signal transduction	124	4.15E–06	0.003134901
GO_BP	GO:0016525	negative regulation of angiogenesis	17	4.78E–06	0.003134901
GO_CC	GO:0005886	plasma membrane	351	3.53E–10	1.80E–07
GO_CC	GO:0070062	extracellular exosome	269	7.23E–10	1.84E–07
GO_CC	GO:0005925	focal adhesion	65	1.73E–09	2.94E–07
GO_CC	GO:0005856	cytoskeleton	59	1.76E–08	2.24E–06
GO_CC	GO:0005604	basement membrane	20	1.89E–06	1.93E–04
GO_MF	GO:0005088	Ras guanyl-nucleotide exchange factor activity	27	5.77E–07	6.69E–04
GO_MF	GO:0005515	protein binding	681	3.95E–06	0.002288774
GO_MF	GO:0008201	heparin binding	28	1.79E–05	0.00691105
GO_MF	GO:0046934	phosphatidylinositol-4,5-bisphosphate 3-kinase activity	16	9.39E–05	0.024528787
GO_MF	GO:0032947	protein complex scaffold	11	1.06E–04	0.024528787
KEGG_PATHWAY	hsa05200	Pathways in cancer	59	2.65E–05	0.006480973
KEGG_PATHWAY	hsa04510	Focal adhesion	35	9.26E–05	0.011337779
KEGG_PATHWAY	hsa04022	cGMP-PKG signaling pathway	27	5.60E–04	0.037355968
KEGG_PATHWAY	hsa04015	Rap1 signaling pathway	33	6.10E–04	0.037355968
KEGG_PATHWAY	hsa05205	Proteoglycans in cancer	31	0.00111396	0.045133137

### Identification of key module in the PPI network

**Figure 3.**
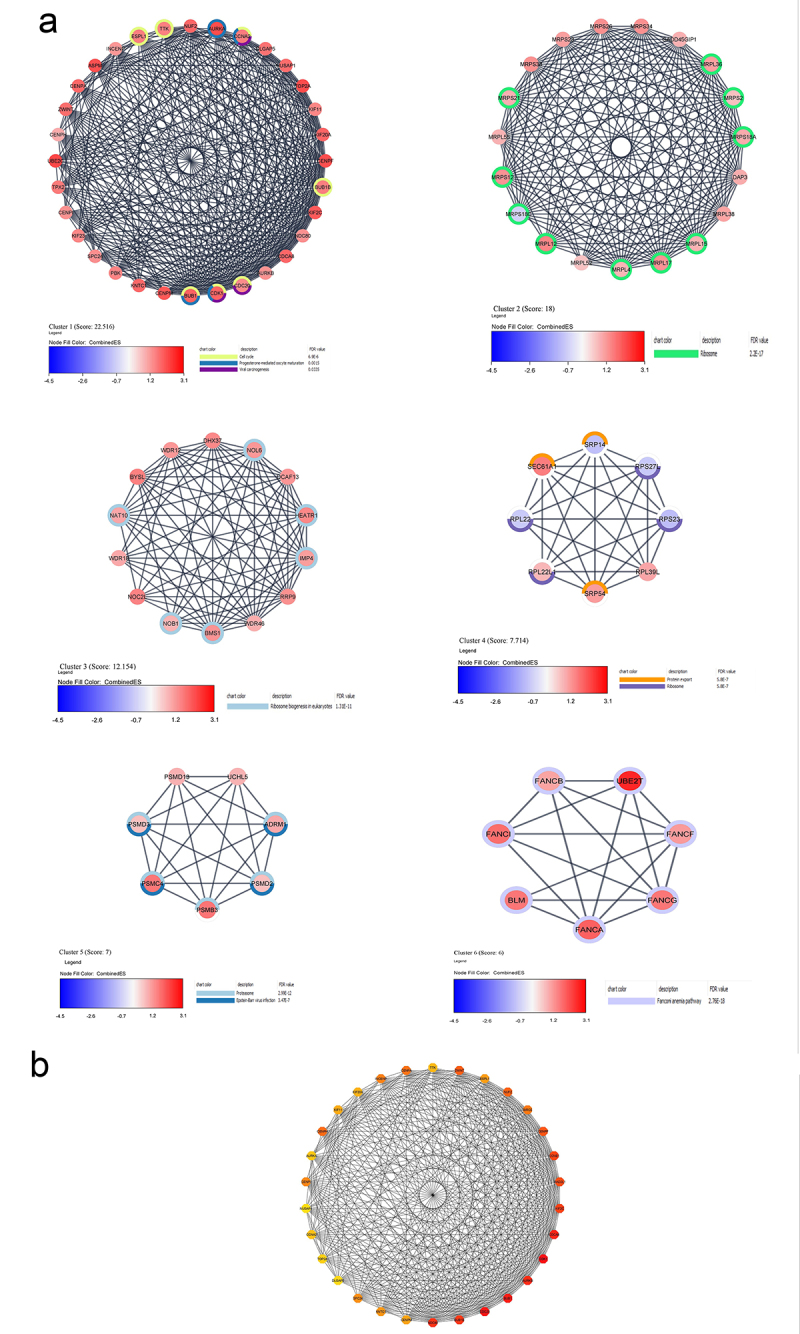
(a) The six main gene clusters and the pathways they are enriched in. (b) 30 hub genes in the PPI network. The color spectrum from red (higher score) to yellow (lower score) indicates the MCC score for each node.

MCODE analysis of the largest PPI network revealed six modules. KEGG enrichment analysis showed that 7 out of 32 genes in cluster 1 are enriched in the cell cycle. Interestingly the DEGs of clusters 2, 3, and 4 were significantly enriched in ribosome and ribosome biogenesis. Most of the DEGs in cluster 5 were enriched in Proteasome and Epstein-Bar virus infection. All 7 DEGs in cluster 6 were enriched in the Fanconi anemia pathway. (Figure 3a showed the 6 clusters and the KEGG enriched pathways).

### Validation and prognosis prediction of hub genes

**Figure 4.**
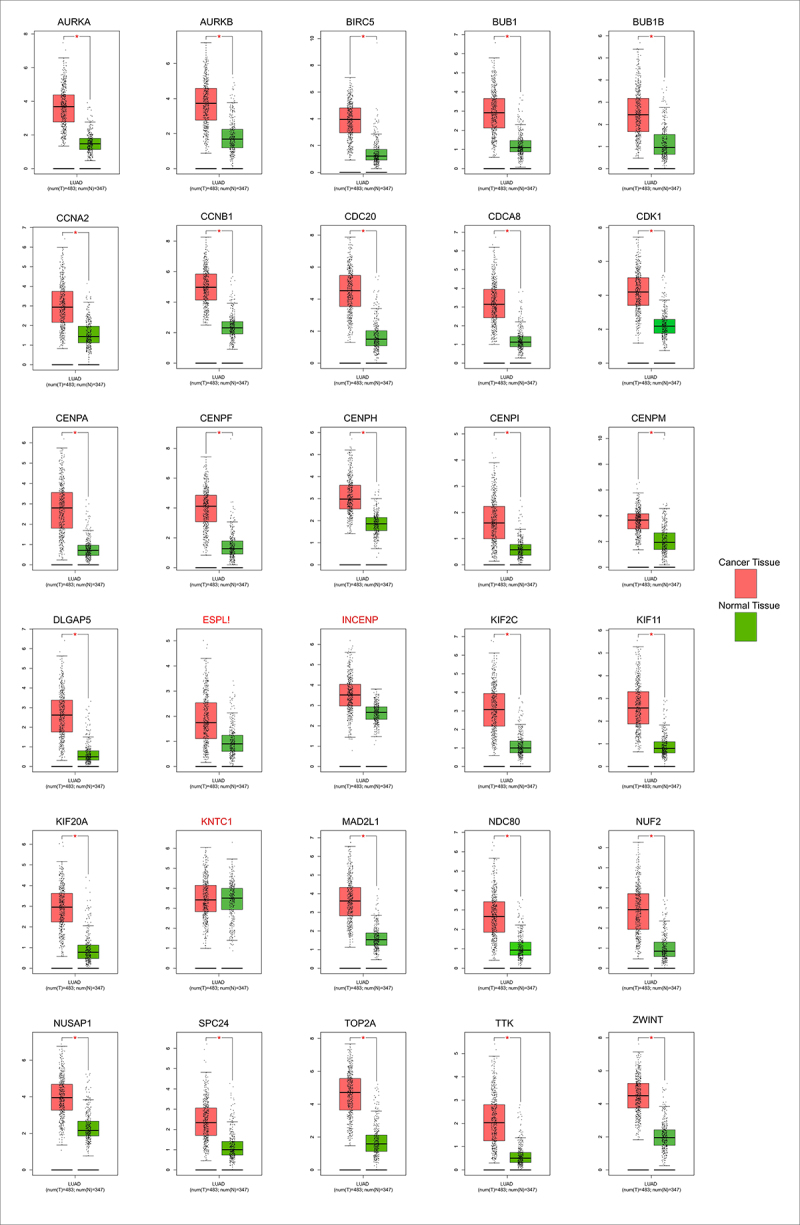
Validation of the expression of the 30 hub genes based on TCGA and GTEx data in GEPIA (http://gepia.cancer-pku.cn). *P<.01. The y-axis unit represents Log_2_(TPM +1). Transcripts per Million (TPM).

**Figure 5.**
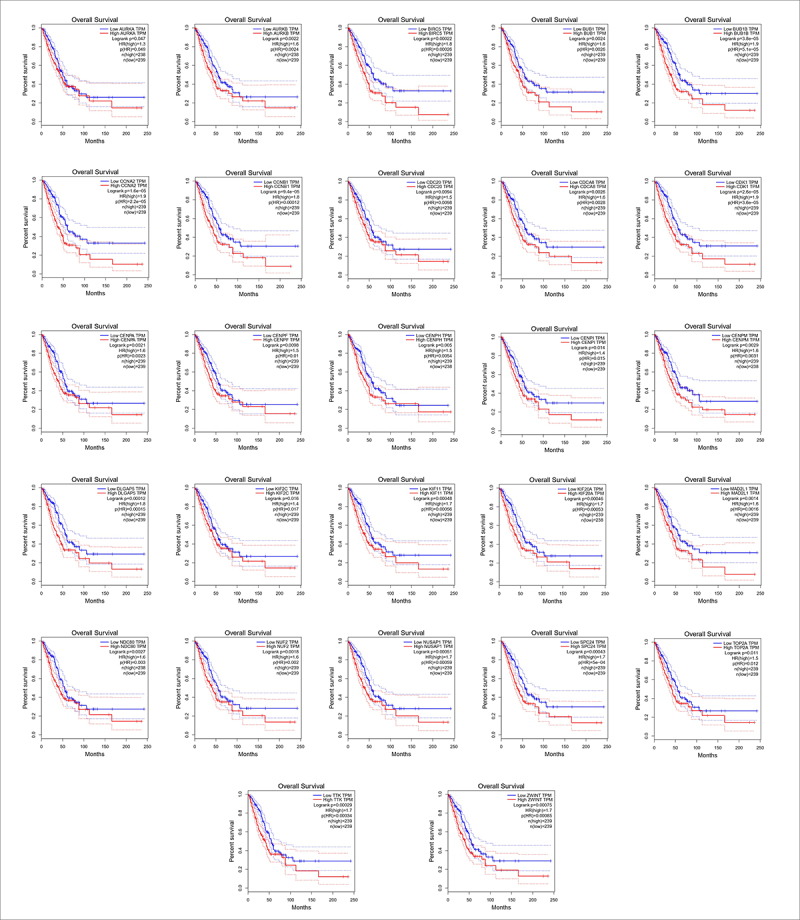
Overall survival and analyses of the 27 validated hub genes were performed using GEPIA.

The differential expression pattern of the 30 most significant hub genes (shown in Figure 3b) selected by CytoHubba were screened using GEPIA. Only three genes (*INCENP, KNTC1*, and *ESPL1*) out of 30 hub genes did not show similar expression patterns based on both TCGA and GTEx databases (Figure 4). The Kaplan – Meier overall survival analysis was done for the rest of the 27 hub genes (Figure 5). *CCNA2*, *CDK1*, *BUB1B*, and *CCNB1* had the worst overall survival outcome.

* The dotted lines represent the 95% confidence interval of the survival probability estimates.

### Drug repositioning based on reversal gene signatures

The top 50 drug perturbations (including 32 unique drugs) that can reverse the LUAD expression pattern were listed after submitting the up and down-regulated genes to the L1000CDS^2^ search engine. Most of the drugs obtained had anti-neoplastic effects via different pathways. The top 10 small molecules’ perturbation details in different cell lines and doses showed in Table 2. The cell line, dose, and time point mentioned in the table are those used for generating the signature by L1000CDS^2^. PP-110 and AS-605240 (quinoline derivative chemical) are phosphatidylinositol-3-kinase (PI3K) inhibitors. PI3K/AKT/mTOR signaling pathway governs various cellular functions such as metabolism, motility, proliferation, growth, and survival which is one of the most commonly dysregulated pathways in human malignancies.^26^ Puromycin is a well-known antibiotic that suppresses protein synthesis by interfering with peptidyl transfer and releasing polypeptide chains from the translating ribosome.^27^ The anti-proliferative and apoptotic effect of Puromycin has been demonstrated in some research.^28,29^ The BMS-536924, which is a dual insulin receptor (IR) and insulin-like growth factor-1 receptor (IGF1R) inhibitor, had also shown anti-proliferative and pro-apoptotic effects in vitro and in vivo.^30^ Fedratinib, a drug mainly used to treat myeloproliferative neoplasm (MPN)-associated myelofibrosis, acts as a competitive inhibitor of protein kinases JAK-1, JAK-2, and FLT3, was another drug with the potential of reversing the LUAD expression profile.^31^ The mitogen-activated protein kinase (MEK) inhibitor Selumetinib and the HDAC inhibitor Vorinostat were the two other probable drugs capable of reversing the transcription signature in LUAD. The Raf-MEK-ERK signaling pathway is overactive in several malignancies, and Selumetinib, which inhibits MEK1 and MEK2, can suppress cell proliferation and promotes pro-apoptotic signal transduction.^32,33^ Vorinostat -the first in a new class of drugs known as histone deacetylase inhibitors- impedes the activity of histone deacetylases HDAC1, HDAC2, and HDAC3 (Class I), and HDAC6 (Class II). HDAC overexpression or aberrant interaction of HDACs to oncogenic transcription factors induces hypoacetylation of core nucleosomal histones in specific cancer cells. By inhibiting histone deacetylase, Vorinostat increases the accumulation of acetylated histones and cell cycle arrest and also apoptosis.^34^

**Table 2.** Top 10 drugs and their detailed perturbation profiles that predict to reverse the expression pattern in LUAD.

Rank	Score*	Small molecule	Drug Bank Accession number	Mechanism of action	Cell-line	Dose	Time
1	0.0562	PP-110	NA	PI3K inhibitor	HT29	22.2um	24.0 h
2	0.0540	Puromycin Hydrochloride	DB08437	protein synthesis inhibitor	VCAP	10.0um	24.0 h
3	0.0523	BMS-536924	NA	IGF-1 inhibitor	A375	11.1um	24.0 h
4	0.0518	Fedratinib (TG101348)	DB12500	FLT3 and JAK inhibitor	MCF7	11.1um	24.0 h
5	0.0514	Selumetinib (BRD-K57080016)	DB11689	MEK inhibitor	A375	80.0um	24.0 h
6	0.0514	Fedratinib (TG101348)	DB12500	FLT3 and JAK inhibitor	HT29	11.1um	24.0 h
7	0.0514	Selumetinib (BRD-K57080016)	DB11689	MEK inhibitor	MCF10A	10.0um	24.0 h
8	0.0510	Vorinostat (Zolinza)	DB02546	HDAC inhibitor	MCF7	10.0um	24.0 h
9	0.0501	BMS-536924	NA	IGF-1 inhibitor	HT29	11.1um	24.0 h
10	0.0497	AS605240	NA	PI3K inhibitor	A375	10.0um	24.0 h

*score is the overlap between the input DEGs and the signature DEGs divided by the input number. NA=Not Available.

**Figure 6.**
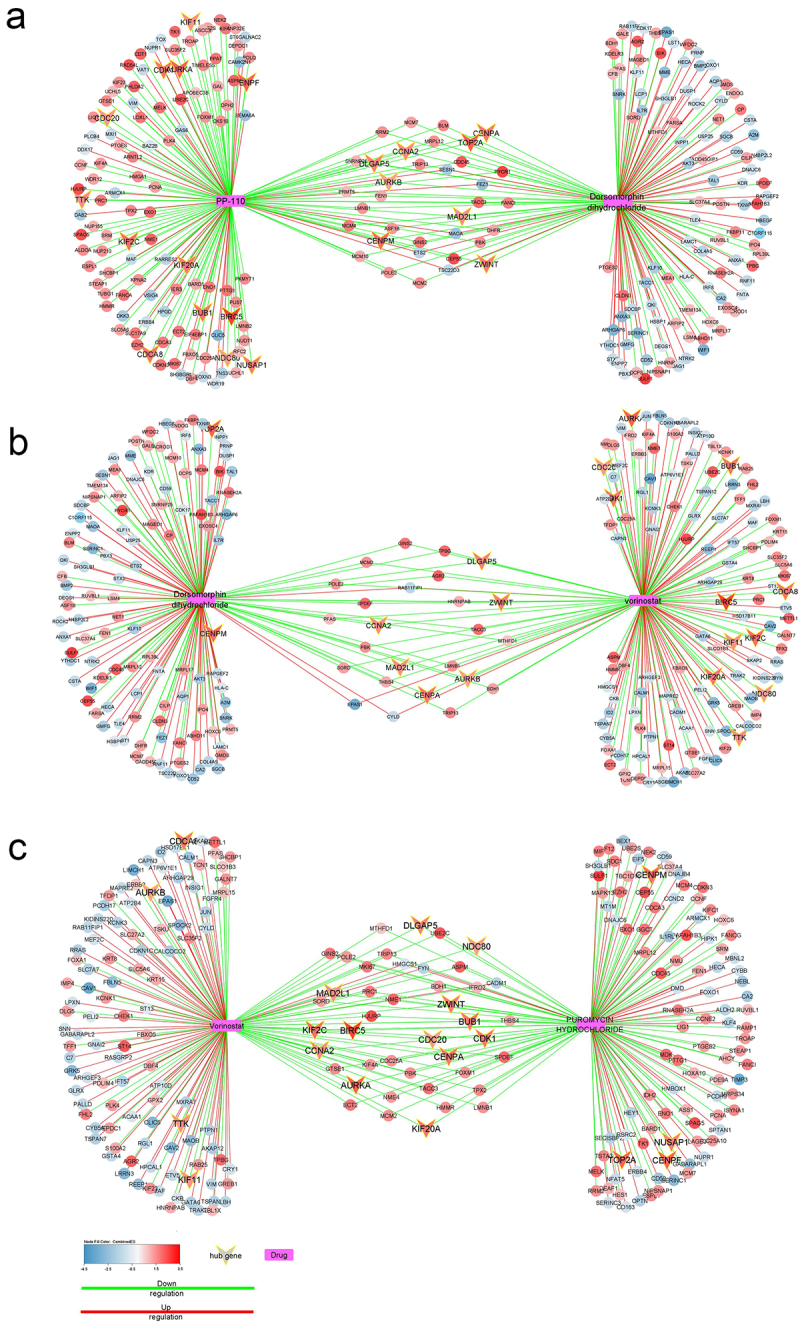
The interactions between the top three drug combinations with the DEGs. Hub genes showed with V shapes. combinedES= combined effect size.

L1000CDS^2^ also provides possible drug combinations. The top 50 drug perturbation signatures were compared, and for each pair, a synergy calculation was performed by combining the overlap of the DEGs produced by the two drug treatments with the DEGs used as input. Table 3 lists the top 10 non-redundant drug combinations. The interactions of the top three drug combinations with the DEGs and hub genes were evaluated (Figure 6). The combined Effect Size (abbreviated as combinedES) is represented in the figure using a color scale. Blue shades indicate a negative effect size, which means that the gene was downregulated in the tumor, while red shades indicate a positive effect size, which means that the gene was upregulated in the tumor. The combination of Vorinostat and Dorsomorphin has been shown to down-regulate 19 of the 27 hub genes. The PP-110-Dorsomorphin combination and the Puromycin-Vorinostat combination can both down-regulate 21 of the 27 hub genes. Dorsomorphin (aka Compound c) is a potent inhibitor of AMP-kinase and BMP type I receptor. Dorsomorphin has been proven in vitro to reduce the viability of glioma and colorectal cancer cells by inhibiting proliferation and triggering cell death. However, the anti-cancer properties of this chemical are attributed to its effect on proteins other than AMPK.^35,36^ Researches indicate several potential anti-cancer properties of dorsomorphin, including up-regulating of BAD (pro-apoptotic protein)^37^ and suppression of DKK1 (Dickkopf-related protein 1)^38^ and HSF1 (Heat Shock Transcription Factor 1),^39^ all of which are carcinogenic when dysregulated.

**Table 3.** Top 10 non-redundant drug combinations with potential synergistic effect.

Rank	Overlap Score *(× 10^−3^)	Drug Combination
1	89.4	Vorinostat – Dorsomorphin dihydrochloride
2	87.7	PP-110 - Dorsomorphin dihydrochloride
3	86.8	Puromycin hydrochloride – Vorinostat
4	86.4	PP-110 - Vorinostat
5	86.4	PP-110 - TW 37
6	86.0	Selumetinib – Dorsomorphin dihydrochloride
7	85.1	Selumetinib – Vorinostat
8	85.1	BMS-536924 - Dorsomorphin dihydrochloride
9	84.7	BMS-536924 - Vorinostat
10	84.7	TW37-Dorsomorphin dihydrochloride

*The overlap score is calculated as the combined overlap of the DEGs of the two drug signatures with the input gene lists.

## Discussion

Lung adenocarcinoma is one of the deadliest cancer subtypes, and various investigations are being undertaken to identify more effective treatments. In this study, we performed a meta-analysis on six different microarray datasets to identify the critical gene clusters and hub genes involved in lung adenocarcinoma and then investigated possible drugs with the potential to reverse the expression of dysregulated genes.

Drugs were classified into two groups: single drugs and drugs with possible synergistic effects. Most of the suggested drugs have already known anti-neoplastic properties. The drugs with the highest scores in the single-drug group demonstrated cancer-specific inhibition features. Puromycin is a known drug with antibacterial characteristics. Puromycin has been shown to halt the process of protein synthesis in tumor cells more than in normal ones.^28^ The other drug, BMS-536924, is an IGF-IR specific inhibitor. The IGF-IR tyrosine kinase regulates several important elements of cellular function. Inhibiting IGF-IR reduces the growth rate of normal cells, while its influence on some cancer cells is strong enough to cause apoptosis.^40^ Fedratinib is a JAK2 and FLT3 inhibitor approved by the US Food and Drug Administration to treat adults with myeloproliferative neoplasms. Fedratinib had favorable outcomes as a synergic therapy in Erlotinib-resistant NSCLC.^41^ Fedratinib seems an ideal tyrosine kinase inhibitor since it did not show any genetic drug resistance by in vitro drug screenings, most likely owing to the fact that it can bind to two distinct locations in the kinase domain, the ATP site and the peptide substrate-binding site.^42^ Another drug in our list of findings, selumetinib, has a good track record for treating NSCLC. Clinical trials showed contradicted our findings, indicating that using this medication alone or in combination with thoracic radiation is ineffective in treating NSCLC.^43^ Despite improved response rates and progression-free survival when combined with docetaxel, it failed to meet pre-specified endpoints in phase III clinical trials.^44^ Vorinostat, a histone deacetylase inhibitor approved by the FDA to treat cutaneous T-cell lymphoma, is the other potential drug. Considering that overexpression of the epigenetic modifiers HDAC1 and HDAC3 is associated with a poor prognosis in lung adenocarcinoma,^45,46^ Vorinostat looks to be a promising therapeutic option. Furthermore, HDAC inhibitors have been shown to cause DNA damage and thus activate apoptotic pathways in cancer cells, while normal cells are typically resistant to HDAC inhibitor-induced cell death.^47^ However, clinical studies employing Vorinostat as a monotherapy revealed an inadequate anti-tumor response in patients with relapsed NSCLC.^47^ The information on the other two drugs, PP-110 and AS605240, is restricted in the literature and databases such as drug bank,^48,49^ DrugCentral,^50^ and Therapeutic Target Database.^51^ They both exhibit anti-tumor effects via PI3K inhibition, according to the iLINCS knowledge base,^52^ but no data on clinical investigations is available.

The complexity and heterogeneity of cancer is a significant barrier to the development of effective anti-cancer treatments. Monotherapies are vulnerable to drug resistance because cancer cells use alternative pathways and mutations to fulfill their survival needs. However, drug combination dramatically reduces the likelihood of drug resistance, as two or more pathways are targeted simultaneously; this results in greater effectiveness, fewer adverse effects, and lower toxicity.^53^ Thus we assessed probable drug combinations with synergic effects.

The top drug combinations on our list comprised four main small molecules: Vorinostat, Dorsomorphin, PP-110, and Puromycin.

We suggest that the combination of Puromycin/Vorinostat is a priority for further investigations, although The PP-110/Dorsomorphin and Puromycin/Vorinostat combinations both interacted with an equal number of hub genes. Vorinostat is the only approved drug among the others, which means it has passed drug safety protocols and has a known pharmacological property, allowing its effectiveness to be evaluated more quickly and at a lower cost. However, the drugs Dorsomorphin and Puromycin are still in the experimental phase, and information on PP-110 is scarce, making it difficult to comment on this drug right now.

In line with our hypothesis, Puromycin has been shown to cause cancer-specific apoptosis via direct interaction with several ribosomal proteins to halt protein synthesis.^28^ Interestingly, our research also indicated that three of the six key clusters associated with lung adenocarcinoma are involved in ribosome biogenesis and protein export. Furthermore, researchers believe that utilizing Vorinostat as a “biologic response modifier” in combination with other anti-cancer drugs is reasonable.^54^ It is also shown that the drug has synergistic advantages with some conventional anti-cancer drugs in both pre-clinical and clinical studies.^55,56^

This study proposed drugs with a greater chance of success in clinical trials by assessing the impact of existing drugs on the expression profile of lung adenocarcinoma.

However, our findings must be seen in light of some limitations. Bioinformatics analysis alone cannot determine which of the two drug combinations will be more effective, especially given the close overlap scores. Further evaluation and validation of the drug combinations are necessary to confirm their synergistic effect in appropriate lung adenocarcinoma cell lines and dose-response experiments must be done to determine the optimal dose range of each drug that achieves the highest synergistic effect. It must be noted that the number of drugs assessed in the LINCS L1000 database is limited; and, there may be a bias in the number of tests performed for each drug that affects our results.

Further research is needed to validate the anti-tumor effects of the proposed drugs on lung adenocarcinoma. Also, the detailed pharmacokinetics of drugs, clinical efficiency, and the safety of drug-drug interactions must be assessed experimentally.

## Data Availability

The microarray datasets that were analyzed during the current study are available in GEO repository (www.ncbi.nlm.nih.gov/geo). Accession numbers are listed in the manuscript.
